# Embedding Synthetic Microvascular Networks in Poly(Lactic Acid) Substrates with Rounded Cross-Sections for Cell Culture Applications

**DOI:** 10.1371/journal.pone.0073188

**Published:** 2013-09-02

**Authors:** Jen-Huang Huang, Jeongyun Kim, Yufang Ding, Arul Jayaraman, Victor M. Ugaz

**Affiliations:** 1 Artie McFerrin Department of Chemical Engineering, Texas A&M University, College Station, Texas, United States of America; 2 Department of Biomedical Engineering, Texas A&M University, College Station, Texas, United States of America; Institute for Frontier Medical Sciences, Kyoto University, Japan

## Abstract

Synthetic microvascular networks are essential to enable *in vitro* studies of cell biology, biophysics, hemodynamics, and drug discovery, as well as in applications involving tissue engineering and artificial vasculature. But current limitations make it challenging to construct networks incorporating a hierarchy of microchannel diameters that possess cell-favored circular cross-sectional topographies. We report a new approach that overcomes these limitations by employing pressure-assisted expansion of biocompatible degradable poly(lactic acid) (PLA) substrates. This single-step process is straightforward and highly controllable, making it possible to simultaneously shape the interior topology of branched 3D and pseudo-3D microchannel networks across wide range of diameters. We further demonstrate *in vitro* culture of confluent endothelial cell monolayers in microchannel networks treated by this process, suggesting potential as a tool to help generate bio-mimicking vascular-like environments.

## Introduction

Physiological systems rely on branched flow networks to achieve efficient transport throughout extended 3D spaces [1], [2], making the ability to replicate these features *in vitro* critically important in many fields [3]–[8]. Planar micromachining is the most mature technology to construct synthetic microvascular networks, but it is not an ideal solution because cross-sectional profiles of the corresponding microchannels are typically square, rectangular, or trapezoidal. These topographies make it challenging to establish fully endothelialized microenvironments due to nonuniform cell seeding in the sharp corner regions [9]. Wall shear stresses can also vary widely at these locations [10], [11], potentially impacting flow-based studies [12]–[16]. Rounded cross-sectional profiles are therefore highly desirable, and recent progress toward this goal has made it possible to construct semi-circular topologies by applying methods such as photoresist-based molding [17], wet etching [18], electroplated molding [19], laser ablation [20], and micromilling [21]. Circular cross-sections are then produced by aligning and bonding two substrates imprinted with complementary semi-circular patterns. Sacrificial approaches also permit circular profiles to be obtained upon embedding and removal of soldering wire [22], sugar fibers [23], and polylactide fibers [24]. Post-fabrication circularization has also been demonstrated by coating rectangular microchannels with solvent diluted poly(dimethyl siloxane) (PDMS) [25]–[27] and poly(methyl methacrylate) (PMMA) [28]. Alternatively, direct-ink writing has been employed to construct networks with circular cross-sectional shapes in hydrogel matrices albeit in a low throughput serial format [29]. In addition to limitations in fabrication technology, material-specific issues also pose challenges to long-term culture and biological response studies, particularly in the case of PDMS which can be susceptible to nonspecific surface absorption and leaching of uncured monomer [30], [31].

## Results and Discussion

**Figure 1:**
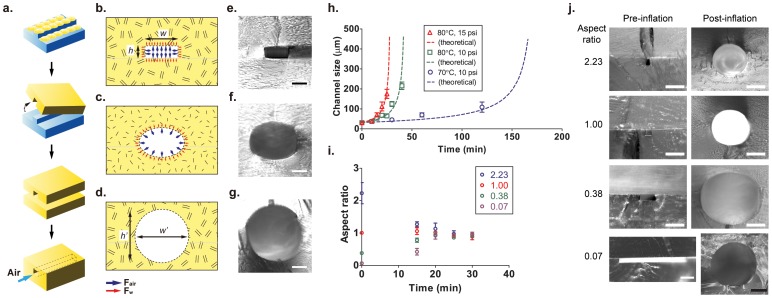
Enlargement and circularization of PLA microchannels by pressure-assisted expansion. (a) Microchannels with initially rectangular cross-sectional profiles are molded in PLA using a PDMS master. (b–d) Pressurized air is injected upon heating the PLA into the rubbery state (i.e., above the glass transition temperature and below the melting temperature) causing the interior air pressure force to exceed the wall resistance. The initially rectangular channels eventually attain circular cross-sections. (e–g) Corresponding images of cross-sectional profiles obtained at different times during the expansion process (*w_0_* = 81 µm, *h_0_* = 31 µm) (e) before expansion; (f) 80°C, 15 psi for 15 min; and (g) 80°C, 15 psi for 25 min (expansion ratio = 5; bars, 50 µm). (h) Comparison between experimental results (symbols) and model predictions (lines) capture the expansion of initially rectangular microchannels as a function of time under different processing conditions (*w_0_* = *h_0_* = 31 µm). Microchannel size is expressed in terms of the instantaneous width (*w*). (i) Circular cross-sectional profiles are obtained regardless of initial rectangular channel aspect ratio (*h_0_*/*w_0_*; 80°C at 15 psi). (j) Cross-sectional images of channels before and after expansion under the same conditions as (i) with initial aspect ratios of 2.23 (*w_0_* = 16 µm, *h_0_* = 35 µm), 1.00 (*w_0_* = *h_0_* = 31 µm), 0.38 (*w_0_* = 81 µm, *h_0_* = 31 µm), and 0.07 (*w_0_* = 498 µm, *h_0_* = 33 µm) (white bar, 100 µm; black bar, 1000 µm). All experiment data are mean ± sd of 3 independent experiments.

We have developed a new strategy to overcome many of these barriers that have hindered previous efforts to construct synthetic microvascular networks. Our approach is based on pressure-assisted expansion of biocompatible degradable poly(lactic acid) (PLA)–a versatile process that can be readily applied in both 2D and 3D branched networks incorporating a hierarchy of microchannel diameters. This treatment simultaneously renders the interior surfaces circular and smooth, yielding a robust cell-favored microenvironment capable of supporting uniform seeding and culture of endothelial monolayers under continuous flow. Rigid microchannels are first constructed in PLA by molding from a negative PDMS template (Fig. 1a). The resulting microchannels with rectangular cross-section are subsequently enlarged and shaped into circular profiles by heating the substrate and injecting compressed air under constant pressure (Fig. 1b–g). When this process is performed in the glassy state, the interior air pressure force (*F*_air_) is counteracted by resistance from the surrounding rigid wall (*F*_wall_) (Fig. 1b,e). Increasing the temperature above the glass transition (*T_g_* ∼ 55–58°C) causes the material to enter a rubbery regime where *F*_air_ overcomes *F*_wall_ (Fig. 1c,f). Incubating a 4 mm thick substrate at 80°C for 8 min is sufficient to allow the interior to thermally equilibrate (confirmed by placing a thermocouple probe between PLA layers; thermal conductivity = 0.13 W m^–1^ K^–1^, specific heat capacity = 2,135 J kg^–1^ K^–1^). Under these conditions, the microchannel initially assumes an elliptical profile, ultimately attaining an enlarged circular cross section after ∼ 20 min (Fig. 1d,g). The process is arrested by quenching the sample at room temperature.

The final microchannel diameter achieved upon air expansion is governed by temperature, pressure, and incubation time. This interplay can be captured by a dynamic equilibrium associated with the internal stress balance 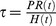, where *τ* is the substrate hoop stress, *P* is the air pressure, *R*(*t*) is the channel radius, and *H*(*t*) is the wall thickness. The time varying wall thickness during enlargement can be approximated as *H*(*t*) = *H_0_–* (*R*(*t*) *– R_0_*), where *H*_0_ and *R*_0_ are the initial values. Assuming the *r*- and *θ*-components of the substrate’s deformation are directly proportional suggests that the strain rate can be expressed as 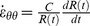, where *C* is an adjustable constant. Equating the hoop stress balance with the corresponding stress component in the *θ*-direction (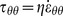, where *η* represents the substrate’s resistance to deformation in the glassy state), yields a differential equation for the time-dependent deformation 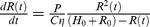. Despite its simplicity (the quantity *Cη* is treated as an adjustable fitting parameter), this model displays remarkably good agreement with our experimental data, capturing the characteristic exponential increase in channel size with time (Fig. 1h, initially square channel cross-sections were considered with *h_0_* = *w_0_* = 2*R_0_* = 31 µm and *H_0_* = 2 mm). Circular cross sections can be obtained from the initially rectangular template microchannels within 20 min over a wide range of initial aspect ratios (*h_0_*/*w_0_* = 0.07–2.23; *h_0_* and *w_0_* are respectively the height and width of the initially rectangular channels) (Fig. 1i), with expansion ratios (*w*/*w_0_*) ranging from 5–10 (Fig. 1j). Elliptical cross sections are produced at shorter times, while bursting eventually occurs in the long time limit.

**Figure 2:**
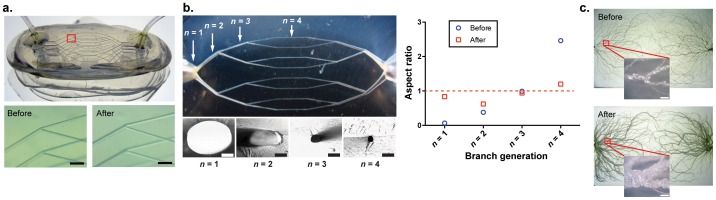
Microchannels throughout a branched network are uniformly expanded and circularized. (a) A pseudo-3D network constructed from a 7 layer stack of planar PLA branched networks before and after expansion (bars, 300 µm). (b) Due to the limitations of 2D lithographic microfabrication, the initially rectangular large channels in early branch generations have smaller aspect ratios than those in later generation branches (*n* = 1: *w_0_* = 498 µm, *h_0_* = 33 µm; *n* = 2: *w_0_* = 81 µm, *h_0_* = 31 µm; *n* = 3: *w_0_* = *h_0_* = 31 µm; *n* = 4: *w_0_* = 14 µm, *h_0_* = 35 µm). The degree of circularity (i.e., aspect ratio *h_0_*/*w_0_* approaching unity) is simultaneously improved across all branch generations after a single expansion step (15 psi of pressurized air for 20 min at 80°C; white bar, 500 µm; black bars, 100 µm). All experiment data are mean ± sd of 3 independent experiments. (c) A 3D branched microchannel network embedded in a 1.5×5×8 cm molded PLA block by electrostatic discharge contains a distribution of microchannel diameters that are not optimal for cell seeding (upper image). After air expansion (lower image), average diameters are significantly increased throughout and the sidewall topology becomes smoother (bar, 500 µm).

We applied the air expansion technique in simplified pseudo-3D constructs using a 7-layer stacked microvascular network embedded in PLA (Fig. 2a). The initially rectangular cross section of all four generations within the branched architecture became circularized during a single expansion step, with all microchannels attaining an aspect ratio close to unity. Our method therefore is not only capable of circularizing the cross sectional profiles, it also enables the process to be simultaneously performed across a broad range of microchannel diameters (∼ 1.5 mm to 50 µm) (Fig. 2b). This is a significant finding because although similar hierarchical architectures incorporating rectangular cross sections can be constructed using conventional lithography and molding approaches [6], [8], [32], [33], the microchannels in every branch generation inherently retain identical channel heights. This topology becomes problematic because internal shear rates become highly nonuniform (e.g., low aspect ratio channels display much higher shear rates in the vertical direction than in the horizontal plane), generating severe internal shear stresses that are physiologically unfavorable. We also applied the air expansion method in a fully 3D tree-like synthetic microvascular network embedded in PLA using electrostatic discharge [34]. The interior microchannels were subsequently deformed, yielding a global increase in channel diameter throughout the network as well as smoother sidewall profiles (Fig. 2c). Unlike liquid etchants, pressurized air penetrates the entire network yielding uniform topographies in each branch. Although the bulk PLA substrates considered here contain negligible porosity [35], the same inflation process could be applied toward tissue scaffold applications by embedding a sacrificial porogen in the PLA matrix during microchannel fabrication and subsequent air inflation.

**Figure 3:**
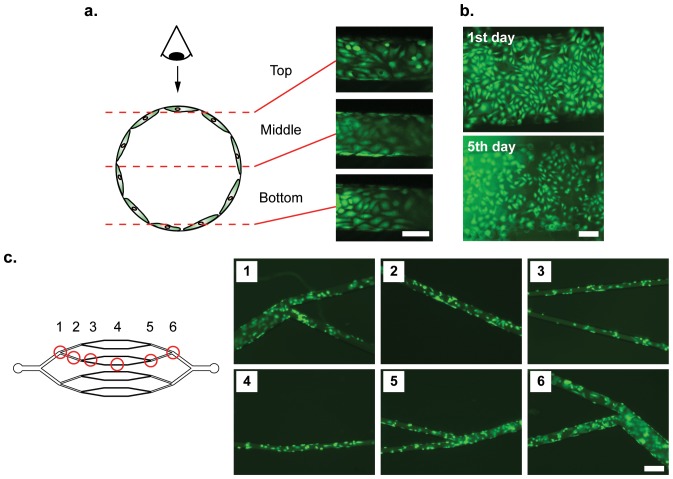
Seeding and culture of bovine aortic endothelial cells (BAECs) throughout PLA microchannel networks. (a) Confocal microscopy shows that the interior walls of a 200 µm diameter straight circularized microchannel can be uniformly seeded with endothelial cells that subsequently are confluently cultured in a monolayer lining the channel wall. (b) Fluorescent images show BAECs survive and maintain their morphology after 5 days in the straight circularized microchannel (bar, 50 µm). (c) BAECs seeded in four generations of branched microchannel network with diameters extending below 50 µm uniformly cover all channel walls and maintain viability after 3 days of culture (bar, 50 µm).

To study cell morphology under dynamic flow conditions during long-term culture, and to demonstrate feasibility of employing PLA substrates for cell-based studies, bovine aortic endothelial cells (BAECs) were loaded with the CellTracker viability dye and seeded within 2D circularized synthetic microvascular networks. We continually perfused culture medium at 0.5 µL min^–1^ through a closed recirculation loop so that cells are continuously exposed to cell-derived growth factors and signaling molecules. Live cell microscopy revealed uniform attachment and growth of a confluent BAEC monolayer along the microchannel walls (Fig. 3a). Cell viability and morphology could be maintained for at least 5 days in straight circularized PLA microchannels under continuous flow conditions (Fig. 3b; Note that due to the long term incubation, CellTracker stained cells displayed weak florescence after day 5).

We also successfully seeded endothelial cells within four generations of a branched microchannel network whose smallest channel size was below 50 µm (Fig. 3c), with viability maintained up to 2 days. We found that the shorter-term viability in the branched configuration reflects unique challenges associated with culture in synthetic microvascular networks as compared with previous studies focused on conventional straight microchannels. First, the hierarchy of channel sizes leads to differences in local cell density within each branch. Consequently, the same seeding conditions may place gaps between adjacent cells in larger channels while simultaneously imposing crowding and overlap in smaller peripheral branches. The less severe sidewall curvature in larger microchannels (>100 µm diameter) is also more favorable to establish uniform coverage, whereas very different environments emerge in smaller capillaries whose dimensions approach those of the flattened cells (∼ 50–100 µm in length, 10–20 µm in width). The ability to successfully seed BAECs throughout an entire branched network is therefore a significant finding because it represents a more physiologically realistic scenario than has been previously considered. More work is needed, however, to identify flow conditions that optimally achieve uniform long-term culture throughout the entire system.

## Conclusions

In conclusion, we have developed a new approach to precisely control microchannel diameters within branched flow networks embedded in the degradable biopolymer PLA based on by pressurized air expansion under conditions near the substrate’s glass transition. This method enables a broad range of channel diameters to be widened throughout an entire network regardless of its complexity, while simultaneously rendering the cross-sectional profiles circular and smooth. The resulting cell-favored microenvironment makes it possible successfully culture endothelial cells within the branched networks–a critical step toward rational construction of synthetic microvasculature.

## Materials and Methods

### PLA Substrate Preparation

Microchannel patterns were photolithographically defined using a transparency film mask printed at 65,024 dpi. The resulting structures were imprinted in SU-8 photoresist (MicroChem) and used as master molds to replicate the inverse microchannel patterns in PDMS (Sylgard 184; Dow Corning; prepared using a 10∶1 degassed mixture of prepolymer and curing agent). After curing for 2 h at 80°C, the cooled PDMS slab was peeled from the master mold, rinsed with 2-propanol (Fisher Scientific), and dried in a stream of compressed air to produce a soft elastomer template. Microchannel patterned PLA substrates were then constructed by directly placing pelletized PLA resin (MW = 1.04×10^5^ g mol^–1^, NatureWorks, grade 3051D; Jamplast Inc.; complete property data are available on the NatureWorks website) onto the PDMS mold and heating to 180°C for 1 h under vacuum, followed by another 1 h at atmospheric pressure and cooling at room temperature for 1 h. After peeling from PDMS mold, fluidic access ports in the PLA microchannel were drilled with a 1/32 inch bit. Smooth planar substrates were obtained by placing pelletized PLA resin directly onto glass microscope slides. A silane pre-treatment was applied to enable easy release of the PLA film by exposing the slides to tridecafluoro-1,1,2,2-tetrahydrooctyltrichlorosilane vapor (SIT8174.0; Gelest Inc.) inside a desiccator for at least 2 h at room temperature. The glass slides containing the PLA pellets were heated to 180°C under vacuum for 1 h, followed by an additional 1 h of heating after the vacuum was released. The slides were then cooled at room temperature for 1 h, after which the PLA film could be easily peeled away. Both the PLA-based molded microchannel and planar substrate were rinsed with 2-propanol followed by DI water and dried with compressed air. Enclosed microchannels were fabricated by bringing the two parts into contact and heating at 80°C for 20 min (the bond strength was not sensitive to the substrate thickness at the size scales of interest here). Tygon tubing (0.03 inch O.D.) was inserted into the access ports and sealed with epoxy glue.

### Expansion-based Deformation

Microchannel networks were coupled with a pneumatic drive system consisting of compressed air, a pressure regulator, and a pressure gauge. The pressurized assembly was heated in a vacuum oven for a prescribed time (VWR 1415M, West Chester, PA), after which the pressure valve was closed and the microchannel was removed from the oven to cool at room temperature. Contraction effects upon cooling are minimized by PLA’s inherently low mold shrinkage (0.004 in/in, data provided by NatureWorks® Co.).

### Modeling the Deformation Process

Matlab software (version 7.10.0) was used to evaluate the time-dependent deformation by solving the differential equation-based model described in the main text. The function *ode45* was applied with initial conditions *H_0_ = *2 mm and *R_0_ = *16 µm under air pressure conditions of 10 and 15 psi. Values of the parameter *Cη* that best fit the experimental data were 5.5×10^6^ Pa·s at 70°C and 1.55×10^6^ Pa·s at 80°C.

### PLA-based Microvascular Networks

Pseudo-3D multilayer branched networks were constructed by stacking 7 patterned PLA microchannels incorporating drilled interconnecting vias on top of a flat substrate, all of which had first been rinsed with 2-propanol followed by DI water and dried with compressed air. The stacked assembly was then thermally bonded at 80°C for 20 min to form an enclosed flow network, after which Tygon tubing (0.03 inch O.D.) was inserted into the open access ports and sealed with epoxy glue. Fully 3D networks were constructed in PLA using an electrostatic discharge process we previously developed [34]. Rectangular PLA blocks were thermally molded from pelletized resin as described above. The substrate was mounted on a carrier tray with 1.5 cm of high-density polyethylene sheets to attenuate the electron beam, and an electric charge was embedded by transporting the carrier trays though a 10 MeV electron beam at a speed of 10 ft min^–1^ at the National Center for Electron Beam Research at Texas A&M. Microchannel networks formed by spontaneous discharge became embedded inside the substrate immediately upon exposure to the beam (holes of 1 mm diameter were pre-drilled to a depth of ∼1 cm, tapering to a point near the bottom of the hole.

### Cell Culture in the PLA Networks

Circularized PLA microchannels were coated with 25 µg mL^–1^ of fibronectin (BD Biosciences) followed by incubation at 37°C in a 5% CO_2_ incubator for 1 h to facilitate the cell attachment. The networks were then rinsed with DMEM medium (SAFC Biosciences) containing 10% FBS (Thermo Science) and 1% penicillin-streptomycin. Bovine Aortic Endothelial Cells (BAECs; Cell Applications, Inc., CA) obtained as a kind gift from Dr. Mariah Hahn at Texas A&M University were resuspended at 5×10^6^ cells mL^–1^ medium for 500 µm diameter channels or at 2×10^7^ cells mL^–1^ medium for 100 µm diameter and branched channels. After inoculation with the cell suspension, the microchannels were placed in the incubator for 2 h to facilitate adhesion. The seeding process was repeated thrice, with the microchannels rotated by 120° each time cells were seeded in the microchannels. After completion of seeding, the attached cells were stained with 25 µM of CellTracker Green CMFDA (Invitrogen) as per the manufacturers instructions. For long term culture, the microchannel networks were interfaced with a computer controlled micropump system and flushed with continuously circulating medium at flow rate of 0.5 µL/min. Cell viability was monitored and imaged using fluorescence microscopy (Axiovert 200M, Carl Zeiss MicroImaging, Inc.).
